# Anti‐asthmatic and anxiolytic effects of *Herissantia tiubae*, a Brazilian medicinal plant

**DOI:** 10.1002/iid3.107

**Published:** 2016-05-01

**Authors:** Talissa Mozzini Monteiro, Hermann Ferrera Costa, Giciane Carvalho Vieira, Paula Regina Rodrigues Salgado, Mirian Graciela da Silva Stiebbe Salvadori, Reinaldo Nobrega de Almeida, Maria de Fatima Vanderlei de Souza, Wemerson Neves Matias, Valdir Andrade Braga, Eugene Nalivaiko, Marcia Regina Piuvezam

**Affiliations:** ^1^ Laboratory of Immunopharmacology, Department of Physiology and PathologyFederal University of ParaíbaJoão PessoaParaíba58051‐970Brazil; ^2^ Department of PharmacyFederal University of ParaíbaJoão PessoaParaíba58051‐970Brazil; ^3^ Department of Biotechnology, Center of BiotechnologyFederal University of ParaibaJoão PessoaParaibaBrazil; ^4^ School of Biomedical Sciences and PharmacyUniversity of NewcastleNewcastleNew South WalesAustralia

**Keywords:** Anxiety, asthma, *Herissantia tiubae*, OVA‐sensitized mice, psychoimmunology

## Abstract

*Herissantia tiubae* (HtE) is a Brazilian plant used in folk medicine to treat inflammatory diseases. Our aim was to determine whether the HtE has anti‐inflammatory and anxiolytic effects in a murine model of asthma. Ovalbumin (OVA)‐sensitized BALB/c mice were treated with HtE (50, 100, or 200 mg/kg) or dexamethasone before each OVA challenge. After the last challenge, animals were subjected to anxiety tests and respiratory measurements. Following euthanasia, we quantified immune cells in the bronchoalveolar lavage (BAL), serum IgE titer and cytokine levels, cellular infiltration and mucus content in the lung tissues, and cellular composition of the mediastinal lymph nodes. OVA challenge in sensitized animals caused: (1) reduction of mean respiratory and dominant respiratory rate (from 398 ± 12 to 286 ± 20 cicles per minute (cpm) and from 320 ± 14 to 162 ± 15 cpm, respectively); (2) increase in behavioral markers of anxiety tests; (3) substantial pro‐inflammatory effects, including rise in OVA‐specific IgE titer (from 0 to 1:2048) and these inflammatory effect diminished the titer to 1:512 after HtE treatment; rise in plasma IL‐13 (from 13 ng/mL in saline to 227 ng/mL in OVA and HtE treatment restored to 1.29 ng/mL; rise in total BAL cell count (from 0.742 cells/mL in saline to 11.77 cells/mL in OVA), with prominent eosinophilia. *H. tiubae* extract affected respiratory parameters similarly to aminophylline, behavioral changes comparable to diazepam, and inflammation being as efficient as dexamethasone. *H. tiubae* extract (HtE) possesses both anti‐inflammatory and anxiolytic properties in the murine model of asthma.

## Introduction

*Herissantia tiubae* [K. (Schum) Brizicky], popularly known in Brazil as “mela bode” and “lava‐prato,” is a shrub belonging to Malvaceaés family; its leaves infusion is used to treat fever and respiratory diseases in folk medicine 1. Two flavonoid glycosides were isolated from aerial parts of *H. tiubae*, kaempherol 3,7‐di‐O‐α‐L‐rhamnopyranoside, and kaempherol 3‐β‐OD‐(6”‐Ep‐cumaroil) 2. Previous studies demonstrated that kaempherol acting on smooth muscle in vascular rings isolated from rat aorta produces vasorelaxant effects 2. Recently, we conducted the toxicological and standardization studies of the leaf extract of *H. tiubae* and demonstrated that the extract is not toxic at dose of 2.000 mg/kg 3.

Despite the established use of *H. tiubae* in folk medicine, there are neither clinical trials for testing its efficacy nor preclinical animal studies for documenting its respiratory effects. Thus our main aim was to test the *H. tiubae* extract (HtE) in a murine model of asthma.

Asthma is one of the most common chronic diseases in the world, and its prevalence increased over the past 50 years to become the most prevalent chronic illnesses in developed countries 4, 5. Asthma is associated with airway mucosal inflammation 6 which increases migration and activation of mast cells, eosinophils, T‐lymphocytes, and macrophages 7, 8. IgE and cytokines such as IL‐4, IL‐5, and IL‐13 are responsible for the persistent chronic inflammation of the airways 8, 9, 10.

One additional and still not broadly recognized aspect of asthma is its association with affective disorders such as anxiety 11, 12, 13, 14, 15. Indeed, research showed that around 80% of asthmatic patients have uncontrolled anxiety 16. Importantly, several animal studies modeling asthma confirmed association of asthma symptoms to higher levels of anxiety 14, 15, 16, 17, 18, 19, 20. While mechanistic understanding of this link is under investigation, it is becoming evident that managing anxiety is important for the asthma patient treatment.

Classes of currently used anti‐anxiety drugs are benzodiazepines and selective serotonin reuptake antagonists (SSRI); both are not ideal as the former provokes drowsiness and may cause dependence while the latter requires 2–3 weeks to the onset of effect. It thus appears that for complex management of asthma with associated anxiety, a drug combining anti‐inflammatory and anxiolytic effects would represent a significant benefit. Based on the results presented above, we suggest that HtE could possess such beneficial combination.Thus, our aim was to determine whether HtE prevents or reduces asthma‐related indices, including anxiety, in a murine model of asthma.

## Methods

### Animals

BALB/c mice (7–8‐weeks‐old) and Wistar rats were used in the experiments. Animals were kept in cages at 25 ± 2°C and 12/12‐h light–dark cycle with free access to water and food. The Animal Facility of the Federal University of Paraiba, Brazil supplied the animals. The Committee for Experimentation on Animal Research of Federal University of Paraiba (CEUA N° 102/13) approved the experimental protocols.

### General experimental outline

**Figure 1:**
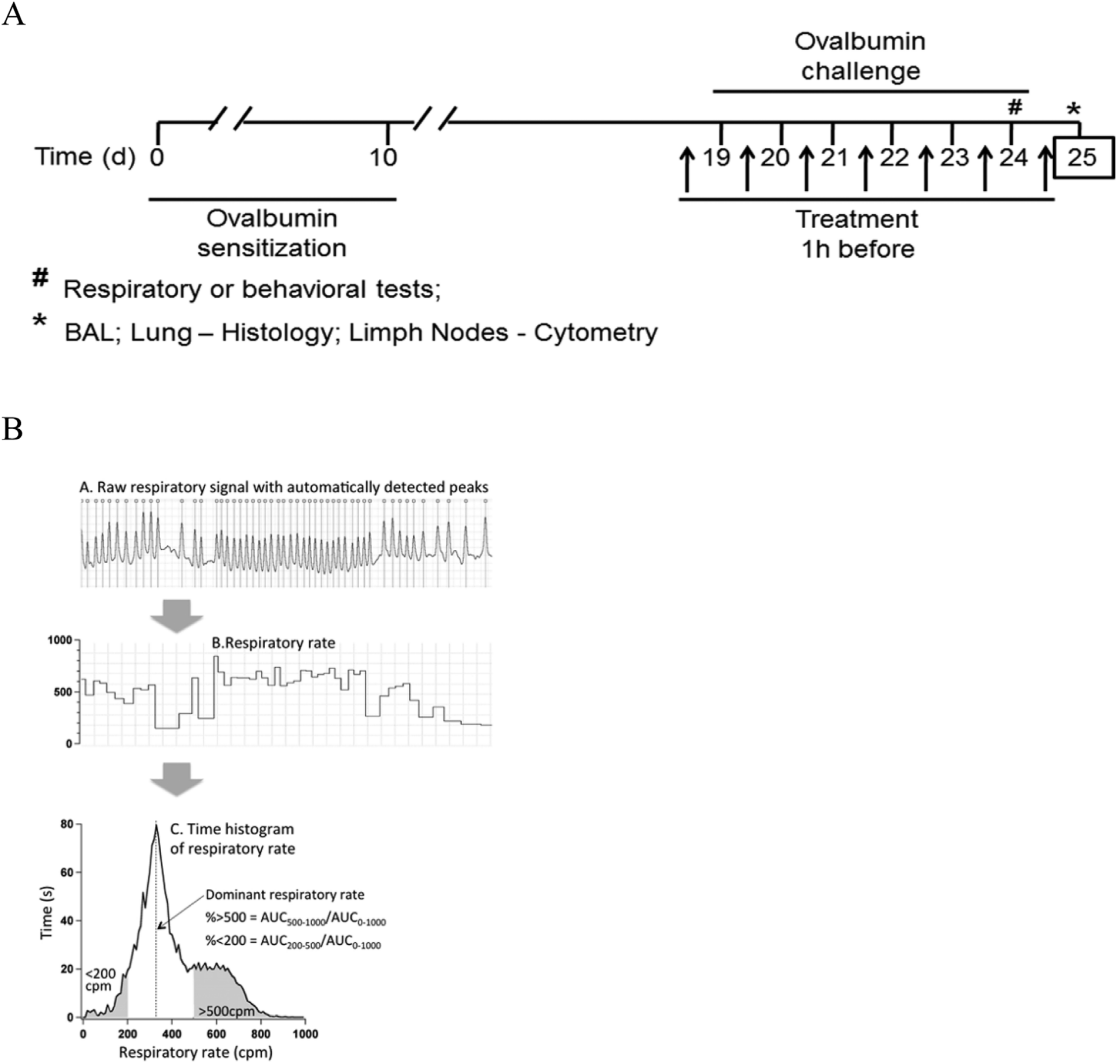
(A) Experimental design; (B) Procedure of respiratory analysis. Top trace—raw respiratory signal with automatically detected peaks. Middle trace—respiratory rate. Bottom graph—time histogram of the respiratory rate intervals. The median corresponds to the dominant respiratory rate.

A schematic of experimental protocol is shown in Figure 1A. Mice were assigned to five groups: (1) Saline group: non‐sensitized mice challenged with ovalbumin (OVA); (2) Allergic group: OVA‐sensitized mice that received saline 1 h before OVA challenges; (3) HtE 50, 100, or 200 groups: animals sensitized and challenged with OVA and received HtE at 50, 100, or 200 mg/kg—p.o. 1 h before OVA challenges. Positive control groups were: (1) Dexamethasone (Dexa) or aminophylline (AMI): animals sensitized with OVA and treated with dexamethasone (2 mg/kg —s.c.) or aminophylline (6 mg/kg—s.c.) 1 h before OVA challenges and (2) Diazepam (DZP): animals sensitized with OVA and treated with diazepam (1 mg/kg—i.p.) 30 min before OVA challenges. Twenty‐four hours after last OVA challenge, mice were euthanized with ketamine and xylazine (100 and 10 mg/kg, i.p., respectively) and tissues were collected for ex vivo analysis (see Table 1).

**Table 1:** Layout and procedures of each group

Groups *n* = 6	Sensitization	Treatment	Challenge	Techniques
Saline	No	Saline	Yes	All tests
Ovalbimin	Yes	Saline	Yes	All tests
HtE 50 mg/kg	Yes	EHt 50 mg/kg	Yes	All tests
HtE 100 mg/kg	Yes	EHt 100 mg/kg	Yes	All tests
HtE 200 mg/kg	Yes	EHt 200 mg/kg	Yes	All tests
Dexamethasone	Yes	Dexa 2 mg/kg	Yes	Immunological tests
Aminophylline	Yes	Amino 6 mg/kg	Yes	Respiratory tests
Diazepam	Yes	DZP 1 mg/kg	Yes	Behavior tests

### Induction of allergic lung inflammation

Mice were sensitized with10 μg OVA (Egg Albumin Grade V, Sigma, St. Louis, Missouri) adsorbed with 2 mg aluminum hydroxide on the first and tenth days and challenged on days 19–24th after primary sensitization with 1% OVA aerosol. The challenge procedure was achieved by placing mice for 20 min into an inhalation chamber connected to an ultrasonic nebulizer (Inalamax NS model S3, Brazil).

### Determination of total and differential cell counts in the bronchoalveolar lavage

The bronchoalveolar lavages (BAL) were obtained to total cell counts using Turk solution in optic microscope. For differential counts, samples were centrifuged at 1000 rpm, at 4°C for 10 min. Cytospin slides from each BAL were stained with Panotic reagent. Each slide was analyzed until the count of 100 cells.

### Respiratory measurements and analysis

Immediately after last OVA challenge, animals were subjected to respiratory recording using whole‐body plethysmography as described previously 21. Respiratory signal was recorded using MacLab‐8s/Chart6 data acquisition system (ADInstruments, Sydney, Australia). The sampling rate was set to 1 KHz. Data were analyzed for 30 min (from fifth to 35th min after the challenge). For characterizing respiratory pattern, we used four parameters: mean respiratory rate (MRR) that was computed from peaks in the respiratory signal; dominant respiratory rate (DRR)—respiratory frequency at which animal spent most of time during recordings; percentage of time spent at high (>500 cpm) respiratory frequency (%HRF), and percentage of time spent at low (< 200 cpm) respiratory frequency (%LRF). For the latter three measures, using IgorPro software (Wavementrics, New Castle, Australia), histograms were constructed for each recording, with a bin width of 10 cpm; an example of such histogram is shown in Figure 1B. This graphic representation indicates how much time (in milliseconds) animals spent at a given respiratory frequency. The mode of such histograms represents dominant respiratory rate whereas %HRF was computed as the ratio AUC_500–800_/AUC_0–800_ (AUC = area under the curve); and the %LRF was computed as the ratio AUC_500–800_/AUC_0–800_. We have arbitrarily chosen 200 and 500 cpm as a cut‐off values for low and high frequencies, respectively (Fig. 1B).

### Elevated Plus Maze (EPM) test

The trial began by placing an animal on the central platform of the maze (Insight®, São Paulo, Brazil) facing an open arm 30 min after administration of diazepam (1 mg/kg—i.p.) or 1 h after oral administration of HtE 50, 100, or 200 mg/kg or vehicle. The number of entries and time spent in open arms of the device were counted during a 5 min period. Animal was considered to have entered into an arm only when all four paws were in that arm 22.

### Hole–Board (HB) test

The procedure used for the HB was modified from File and Wardill 23 where each mouse was placed in the center of the HB and allowed to freely explore the apparatus for 5 min. Number of head dips, time spent until the first head dip (latency), and the locomotion were recorded for analysis. A head dip was scored when the head was introduced into the holes at least to the level of the eyes.

### OVA‐specific IgE titer determination

The OVA‐specific IgE titer was determined using the passive cutaneous anaphylaxis (PCA) test as following: 50 μL of serum from each mouse was injected s.c. on the back of a rat. After 48 h, all rats were anesthetized, and 0.5 mL of a solution containing the Evans Blue (1%) and OVA (2.0 mg) was injected into the tail vein. After 30 min, animals were euthanized, and the OVA‐specific IgE titer was determined as the highest serum dilution giving a 5 mm diameter blue flare 24.

### Flow cytometry analysis

For these studies, bronchoalveolar cells (1 × 10^6^/mL) were incubated with anti‐mouse CD3 FITC conjugate and B220 PE (R&D Systems, San Diego, California) for 30 min at 4°C. Cells were washed twice with cold PBS and resuspended in PBS; 10.000 events were acquired using a Becton Dickinson FACScan, and the data were analyzed using WinMIDI software. Cell populations were identified according to criteria described by Van Rijt and colleagues 25. To assess Treg cells were used antibodies anti‐FOXP3 (BD Biosciences Pharmingen), anti‐CD4 and anti‐CD25 (Santa Cruz Biotechnology). Cells from lymph nodes were fixed with paraformaldehyde 2% for 10 min at 37°C and permeabilized in ice with 90% methanol for 15 min. Before labeling, the cells were washed three times in HBSS and incubated with antibodies for 20 min. At the end of incubation, cells were washed and suspended in HBSS for further analyses on flow cytometer.

### Measurement of cytokines

Broncoalveolar lavage from different animal groups was analyzed for the cytokine production. IL‐13, IL‐10, IL‐17, and IFN‐γ were detected by ELISA according with the manufactory instructions (eBioscience, San Diego, California). Cytokine concentrations in the BAL were determined from a standard curve using recombinant cytokines (regression coefficient, *r* ≥ 0.980) (data not shown).

### Lung histology

After obtaining BAL, lungs were inflated through the heart with saline and the largest lobe of the left lung were removed and fixed with 10% buffered formalin for 24 h at room temperature. The fixed tissues were embedded in paraffin and tissue sections, 5 μm thick, were affixed to microscope slides and deparafinized. The slides were stained with hematoxylin‐eosin (H&E) and periodic acid‐Schiff (PAS) and examined under light microscopy (Motic BA 410). Digital photographs were captured by the camera Moticam 5.0 MP. Histology analysis was performed with the software ImageJ (Fiji version 1.46r, a public domain, Java‐based image processing program developed at the National Institutes of Health for the Macintosh).

### Statistical analysis

Data were analyzed by Student's *t*‐test or ANOVA followed by Newman Keuls or Tukey post‐test using GraphPad Prism statistical analysis and graphing software (GraphPad, San Diego, CA). Values given are means ± S.E.M. from at least six animals in each group. A value of *P* < 0.05 was considered significant.

## Results

### HtE effect on respiration

**Figure 2:**
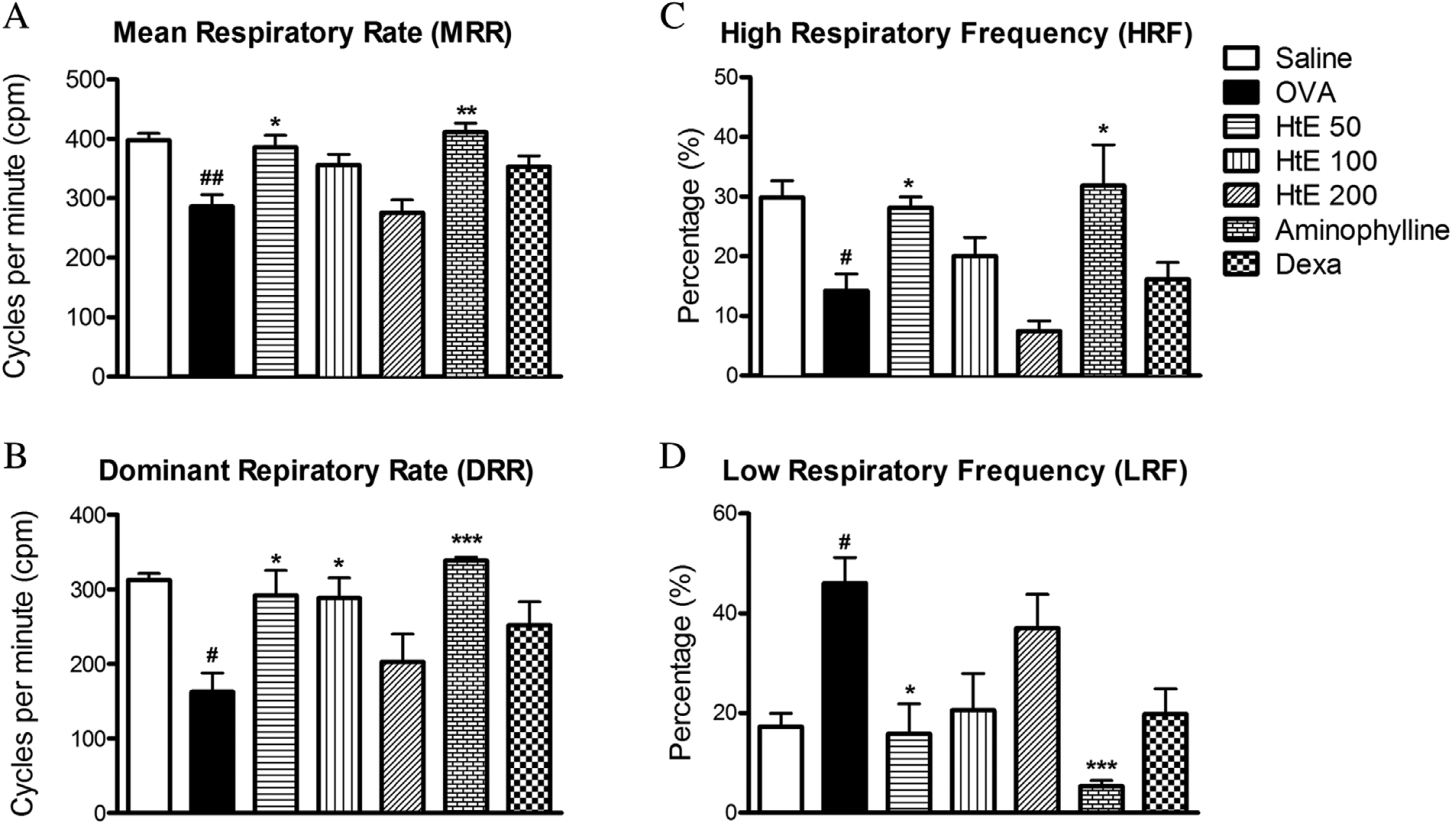
Effect of *Herissantia tiubae* extract (HtE) on respiration. OVA‐sensitized and challenged mice (*n* = 6 per group) were treated with HtE (50, 100, or 200 mg/kg), saline, aminophylline (Amino), or dexamethasone (DEXA) 1 h before each challenge. Immediately after the last challenge, animals were analyzed by plethysmography. (A) Mean Respiratory Rate (MRR); (B) Dominant Respiratory Rate (DRR); (C) High Respiratory Frequency (HRF); (D) Low Respiratory Frequency (LRF). Data are presented as mean ± S.E.M. *P < 0.05 significant compared to OVA group; ^#^ *P* > 0.05 significant compared to saline group.

Animals from OVA group had significantly lower mean and dominant respiratory rate, and spent significantly and substantially more time at low (<200 cpm) respiratory rate and substantially and significantly less time at high (>500 cpm) respiratory rate (Fig. 2). Pre‐treatment with HtE at the lowest dose (50 mg/kg) completely prevented these effects. The two higher doses of the extract were less efficient. Respiratory effects of allergic challenge were also prevented by treatment with aminophylline (Fig. 2). Of note, the drug had reciprocal influence on the low‐frequency and high‐frequency respiratory component, reducing the former and enhancing the latter compared to the allergic challenge alone (OVA group). Pre‐treatment with dexamethasone tended to reverse mean and dominant respiratory rate, without any major effects on the two other respiratory indices (Fig. 2A–D).

### HtE effect on behavior and anxiety

**Figure 3:**
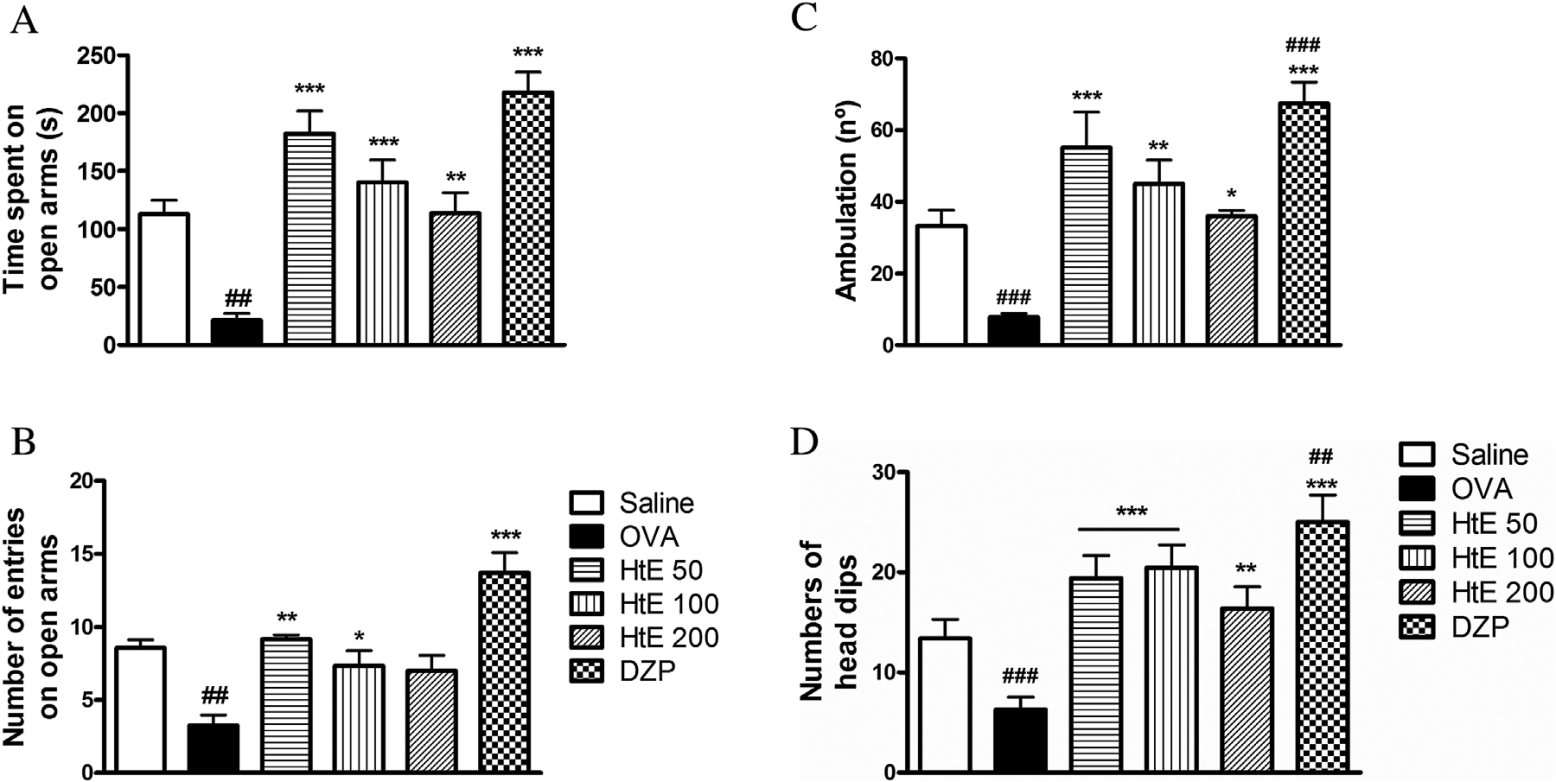
Effect of *Herissantia tiubae* extract (HtE) on anxiety behavior. Ovalbumin‐sensitized and challenged mice (*n* = 8 per group) were treated with the HtE (50, 100, or 200 mg/kg), saline, dexamethasone, or diazepam (DZP) 1 h before each challenge. Immediately after the last challenge, animals were analyzed by both anxiety tests: Elevated Plus‐Maze test (A, B) or Hole–Board test (C–E). (A) Time spent on Open Arms (OA); (B) Number of entries on OA; (C) Ambulation; (D) Latency; (E) Number of Head Dips. Data are presented as mean ± S.E.M. *Significant compared to OVA group; ^#^significant compared to saline group.

Animals from OVA group in Elevated Pluz Maze test showed significant reduction in both time spent in the open arms (OA) and in the number of entries to OA when compared with animals from saline group (Fig. 3A–B). Animals treated with diazepam showed a significant increase in time spent in OA when compared with saline group. Animals treated with HtE (50, 100, or 200 mg/kg) showed a significant increase in time spent in OA, when compared with the OVA group and this increase was similar to those observed in the DZP group (Fig. 3A and B). Number of entries was increased only with HtE treatment at dose 50 mg/kg when compared to the OVA group (Fig. 3A and B).

In the Hole–Board test, animals from OVA group showed significant decrease in locomotion and number of head dips, and increased latency compared to the saline group (Fig. 3C–E). Animals treated with HtE (50, 100, or 200 mg/kg) showed a significant increase of ambulation and the number of head dips, and significantly reduced latency to the first dip compared to the OVA group. Animals from diazepam group showed enhancement of all parameters. Effect of HtE 50 mg/kg was similar to that of diazepam (Fig. 3C–E).

### Effect of HtE treatment on the OVA‐specific IgE serum titer

**Figure 4:**
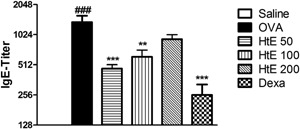
Effect of *Herissantia tiubae* extract (HtE) on OVA‐IgE serum titer. Ovalbumin‐sensitized and challenged mice (*n* = 6 per group) were treated with the HtE (50, 100, or 200 mg/kg), saline or dexamethasone (Dexa) 1 h before each challenge. On the day 25th, animals were euthanized and serum obtained and used to measure OVA‐specific IgE titer (*n* = 10 Wister). Data are presented as mean ± S.E.M. One‐way ANOVA followed by Tukey test. *Significant compared to OVA group; ^#^significant compared to Saline group.

HtE treatment (50 or 100 mg/kg) significantly decreased OVA‐specific IgE serum titer in OVA‐sensitized animals. However, HtE at 200 mg/kg did not change levels of OVA‐specific IgE (Fig. 4).

### Anti‐allergic effect of HtE in asthma models

**Figure 5:**
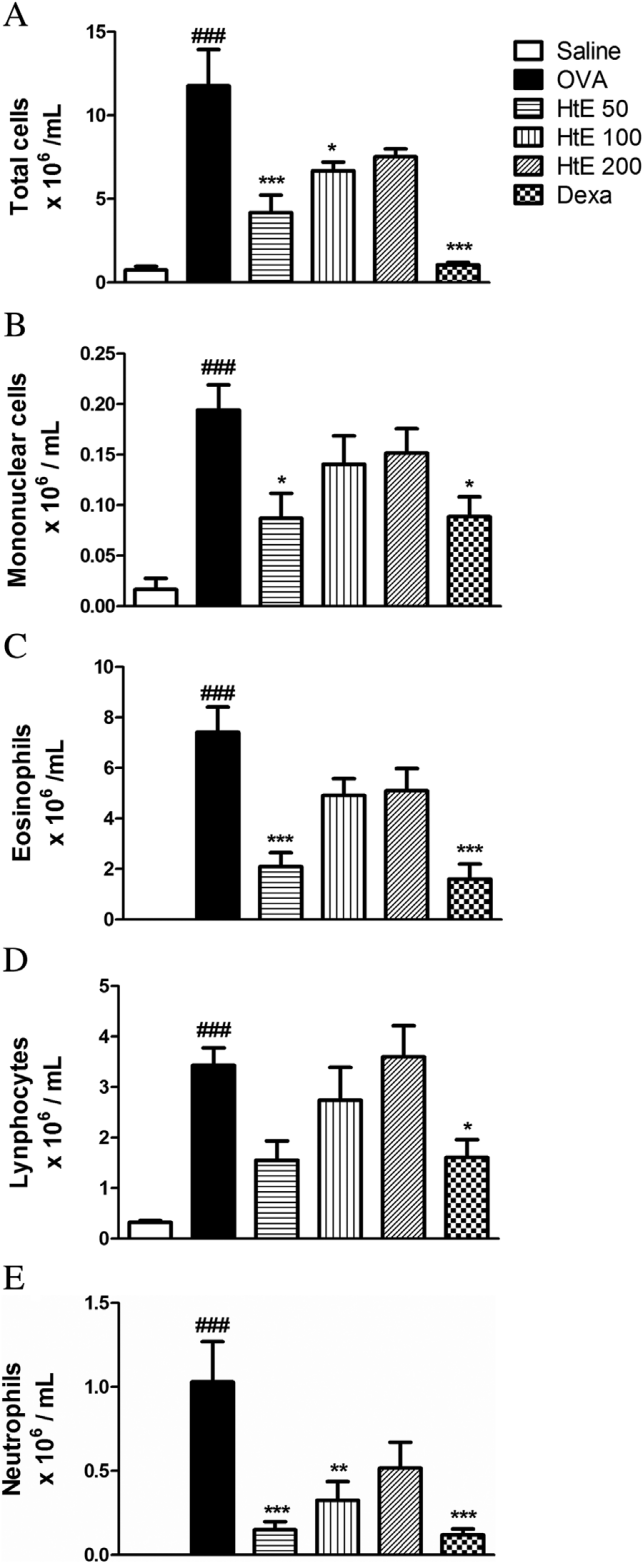
Effect of *Herissantia tiubae* extract (HtE) on inflammatory cells migration to lungs. Ovalbumin‐sensitized and challenged mice (*n* = 6 per group) were treated with HtE (50, 100, or 200 mg/kg), saline or dexamethasone (Dexa) 1 h before each challenge. On day 25th, animals were euthanized and BAL was obtained and used in counts of total and differential cells from all groups. (A) total cells; (B) monocyte; (C) eosinophil; (D) lymphocyte; (E) neutrophils. Data are presented as mean ± S.E.M. (One‐way ANOVA followed by Tukey test and Student *t*‐test). ***P > 0.0001 significant compared to OVA group.

Aerosol allergic challenge of actively sensitized mice induced a marked recruitment of cells to the BAL as evidenced by an intense eosinophil, mononuclear, and neutrophil accumulation (Fig. 5). In addition, oral treatment with HtE at dose of 50 mg/kg (specially) was able to inhibit BAL eosinophil, neutrophil, and mononuclear influx caused by allergic challenge in actively sensitized animals. Furthermore animals treated with standard drug dexamethasone showed similar reduction in total and differential cells as HtE (50 mg/kg) treatment (Fig. 5).

### Effect of oral HtE treatment in different inflammatory cell population

**Figure 6:**
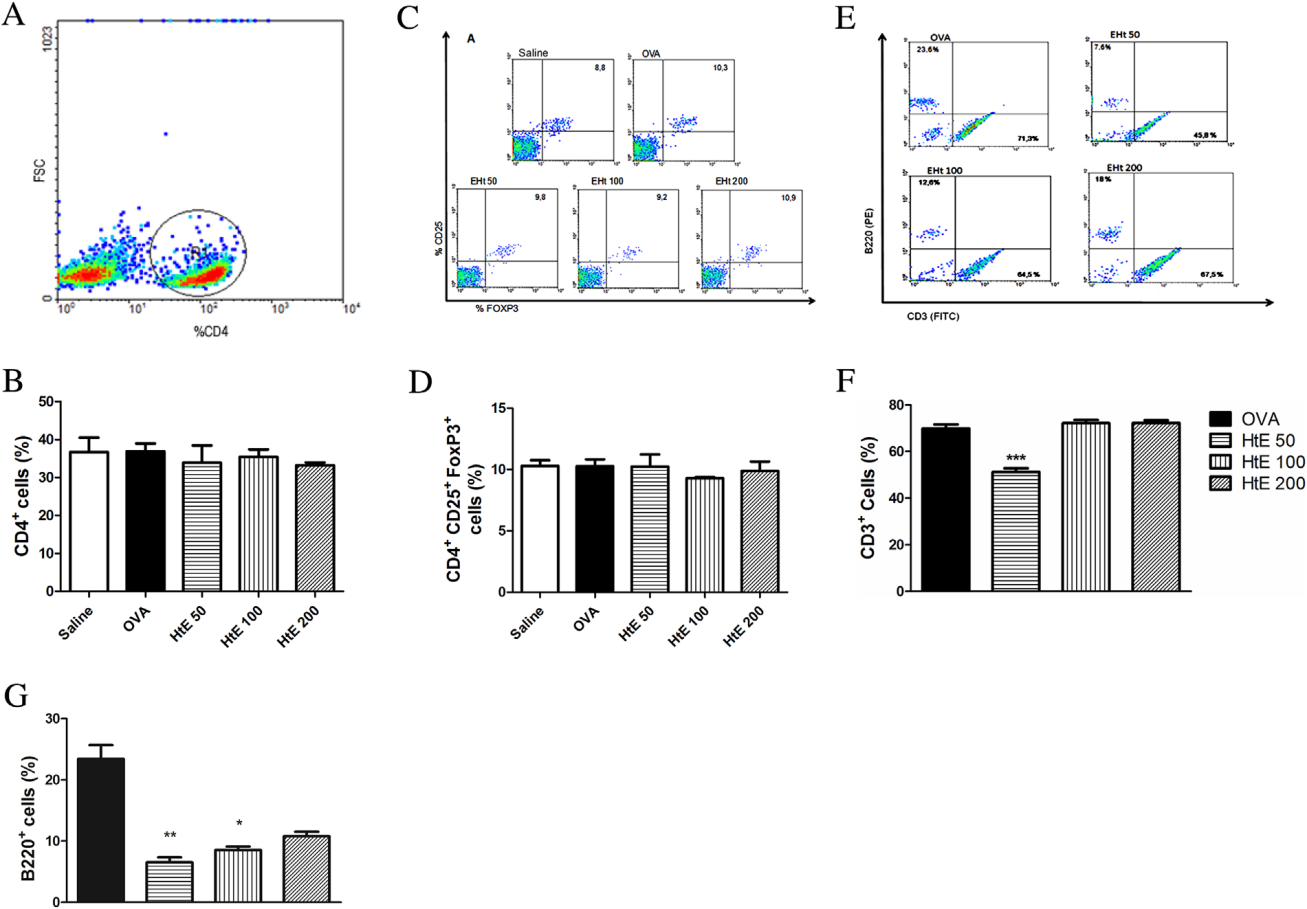
Effect of *Herissantia tiubae* extract (HtE) on lymphocyte subpopulations. Ovalbumin‐sensitized and challenged mice (*n* = 6 per group) were treated with HtE (50, 100, or 200 mg/kg) or saline 1 h before each challenge. On day 25th, animals were euthanized to obtain lymph nodes (Treg cells—results A–D) and BAL (lymphocytes T and B—results E–G) to evaluate lymphocyte populations by cytometer. (A) Dotplot showing the gate; (B) percentage of CD4^+^ cells from lymph nodes; (C) dotplot from lymph nodes of each group tested showing marked cells with FOXP3; (D) percentage of FOXP3 cells in lymph nodes; (E) dotplot OVA and HtE groups at doses of 50, 100, and 200 mg/kg; (F) percentage (%) of labeled B220; (G) Percentage (%) of cells labeled with CD3. Data are presented as mean ± S.E.M. (One‐way ANOVA followed by Tukey test and Student *t*‐test). *Significant compared to the OVA group.

Mediastinal lymph node cells from groups: saline, OVA, and HtE at doses of 50, 100, or 200 mg/kg were stained with CD4^+^CD25^+^ and FOXP3 to assess whether treatment with HtE would be able to change the percentage of Treg cells. The gate was made in a population of lymphocytes labeled for CD4^+^ (Fig. 6A and B). Figure 6C and D shows the tags analyzed after the gate on CD4^+^ lymphocytes, with the upper right quadrant representing doubly labeled CD25^+^ FOXP3 cells. Animals treated with HtE at 50, 100, or 200 mg/kg did not alter the percentage of Treg cells in mediastinal lymph nodes compared to OVA group (Fig. 6A–D).

BAL cells from OVA and HtE (50, 100, or 200 mg/kg) groups were labeled for CD3^+^ and B220^+^ to evaluate the effect of extract on T or B lymphocytes (Fig. 6E). Animals from HtE (50 mg/kg) showed a significant reduction (*P* < 0.001) of TCD3^+^ compared to OVA group (Fig. 6F). However HtE at doses of 100 or 200 mg/kg were not effective in reducing this cell population. Regarding B220 cell population (CD45^+^), treatment with HtE (50 or 100 mg/kg) significantly decreased (*P* < 0.001 and *P* < 0.05, respectively) this cell population in BAL compared with OVA group (Fig. 6G).

### Effect of HtE on cytokines production

**Figure 7:**
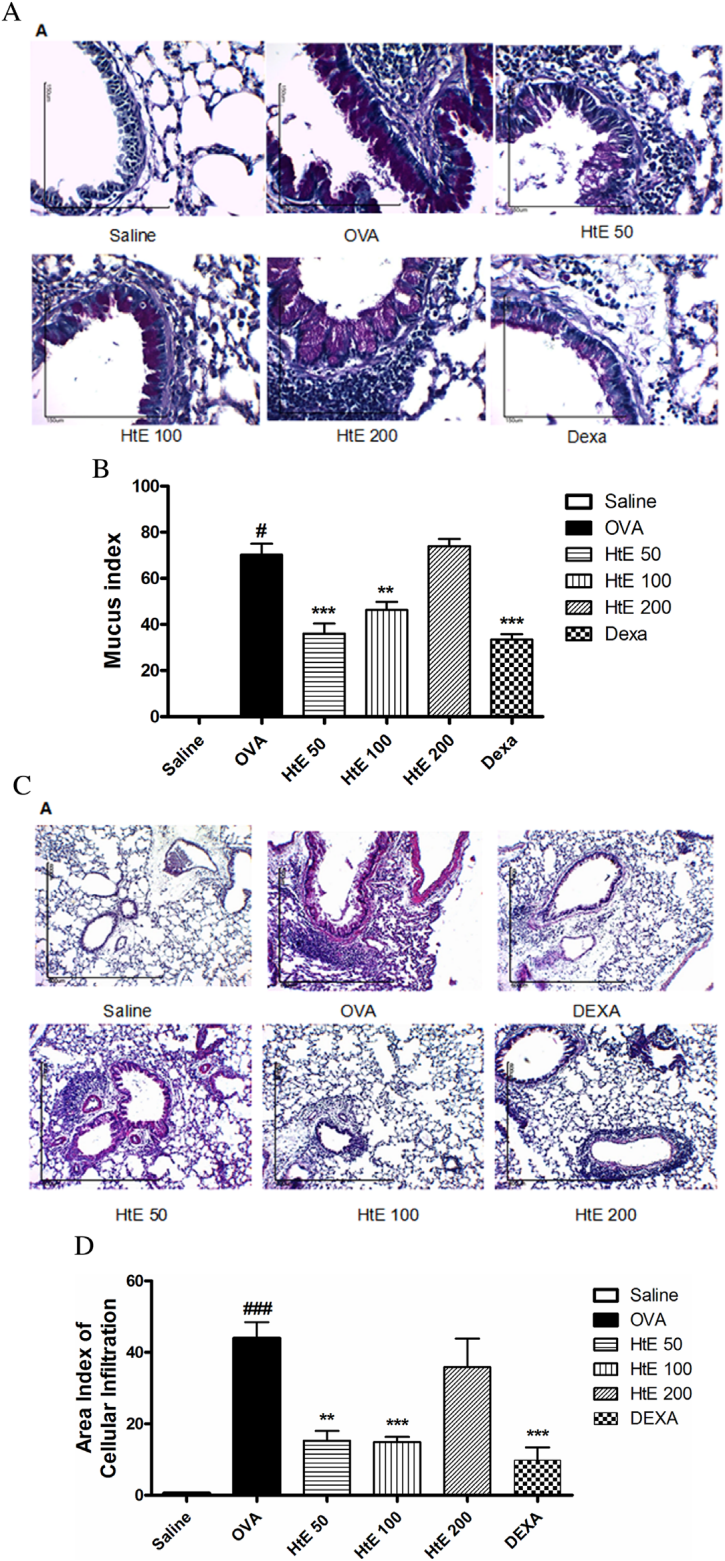
(A) Photomicrograph representative of the airways and lung parenchyma stained with periodic acid Schiff (PAS) in AT 400× captured by camera Motic 5.0 coupled to an optical microscope (*n* = 6 per group). The images were analyzed by ImageJ program using 600‐micron scale bar from Saline, OVA, HtE 50, HtE 100, HtE 200, and Dexa groups, respectively. (B) Statistic analyses. (C) Photomicrograph representative of the airways and lung parenchyma stained with Eosin (HE), observed in a 10× objective and captured by camera Motic 5.0 coupled to an optical microscope (*n* = 6 per group). The images were analyzed by ImageJ program using 600‐micron scale bar. (D) Statistic analyses. Data are presented as mean ± S.E.M. (One‐way ANOVA followed by Tukey test and Student *t*‐test). *Significant compared to the OVA group; ^#^significant compared to the Saline group.

BAL from OVA‐sensitized animals presented high levels of IL‐13 and treatment with HtE (50, 100, or 200 mg/kg) reduces this secretion (Fig. 7D). The reduction was also observed in dexamethasone. Animals treated with HtE in all doses tested did not alter cytokines such as IFNγ, IL‐17, and IL‐10 compared with control groups (Fig. 7A–D).

### Effect of HtE treatment in the lung

After BAL procedure, lungs were removed and taken to histology. Lungs from OVA group showed mucus production by the unicellular exocrine glands (goblet cells) in the primary, terminal, and respiratory bronchioles, while saline group (Fig. 7A) did not present mucus producing cells. Treatment with HtE (50 or 100 mg/kg) inhibited mucus production in terminal and respiratory bronchioles, similar to DEXA group (Fig. 7A and B). Lungs from OVA group (Fig. 7C and D) showed a massive cell infiltration in the peribronchiolar and pulmonary perivascular regions, but absence of cell infiltration in alveoli and vein/venular peribronchiolar congestion, characterizing the pulmonary inflammatory process, while saline groups (Fig. 7C) did not present changes in lung histology. Treatment with HtE (50 or 100 mg/kg) decreased cell migration into peribronchiolar and perivascular regions, similar to DEXA group (Fig. 7C and D).

## Discussion

This study presents the evidence that the leaf extract of *H. tiubae* (Malvaceae), a Brazilian traditional medicinal plant has anti‐inflammatory and anxiolytic effects in murine model of asthma. The extract was assessed for its ability to prevent respiratory, behavioral, and inflammatory symptoms.

In discussing our respiratory data, it must be acknowledged that removing mice from their home cages and placing them into the plethysmographic chamber represented an element of novelty. Any unexpected and new situation evokes active exploratory behavior; in rodents that rely mainly on their olfaction as a primary sensory channel, this exploratory behavior manifests as vigorous sniffing 21, 26. Thus, even in saline group, the respiratory indices most likely were affected by the influence of novelty.

There are at least two different ways whereby allergic challenge could affect respiratory measures. Firstly, similar to previous reports 27, 28, 29, we found extensive mucus secretion in the airways of challenged animals. The resulting increase in the respiratory resistance would prevent the fast‐frequency breathing/sniffing due to inability to rapidly inflate/deflate the lungs. Such slow forced breathing is typical for patients with obstructive pulmonary diseases, and especially during acute asthma attacks. Indeed, we observed that in OVA‐challenged mice, mean respiratory rate was lower compared to Saline group; this shift was caused by reciprocal decrease in fast‐frequency component (reflecting reduced time in a sniffing mode) and increase in a slow‐frequency component (likely reflecting forced breathing). Our respiratory findings are in accord with the only other animal study where respiration was assessed in the conscious state after allergic challenge 30. An alternative (or rather additional) interpretation of our respiratory results is related to the finding that allergic animals scored higher in the anxiety tests. The link between anxiety and respiration is well documented in humans 31, 32, 33, 34, but animal data related to this subject are very limited. The only rodent study focused on the issue reports that in a new environment, more anxious rats have higher dominant respiratory rate but reduced exploratory sniffing compared to low‐anxiety conspecifics 26. Consequently, reduced time in the high‐frequency (sniffing) mode observed in our allergic mice could be the result of the anxiety state provoked by the allergic challenge.

It is difficult to conclude which of the two contributing factors (increased respiratory resistance or increased level of anxiety) dominated in affecting respiratory parameters. The only suggestion that we can make is based on the cited work in high‐ and low‐anxiety rats 26. The fact that in our allergic mice the dominant respiratory rate was actually lower compared to control, even if they had higher anxiety levels, may indicate that the increase in the respiratory resistance was a dominant factor. Pre‐treatment with HtE completely prevented effects of allergic challenges on mean and dominant respiratory rate, and on the fraction of time spent at low‐frequency breathing. These actions of the extract could be attributed both to its anti‐inflammatory effect leading to the reduction of bronchial obstruction and to its anxiolytic properties (see below).

Pre‐treatment with aminophylline restored all respiratory parameters affected by the allergic challenge. The drug possesses several mechanisms of action that could contribute to this effect. Firstly, its direct bronchodilator properties likely led to the reduced respiratory resistance. The latter could have also been diminished by the anti‐inflammatory action of aminophylline. The potent effect of aminophylline is in favor of the idea that the allergy‐related respiratory effects were largely due to an increase in the respiratory resistance. Interestingly, in animals treated with aminophylline, slow‐frequency respiratory component was substantially reduced not only compared to allergic group but also compared to controls. This could be potentially explained by central stimulatory effects of xanthines, including stimulatory action on the respiratory central pattern generator 35.

Results of this study demonstrate that OVA animals rated higher in anxiety tests compared to the Saline group. Similarly to respiratory results, interpretation of this behavioral data is complicated by the fact that they could be a consequence of respiratory obstruction, a general sick state, or both. Sickness behavior is characterized by decreased locomotion, environment exploration, and self‐cleaning 36. Anxiolytic drug diazepam reversed all behavioral markers of anxiety in OVA‐challenged animals. As there is no evidence for the possible beneficial effects of diazepam on the sickness behavior in asthma, this suggests that the principal cause of anxiogenic effect of allergic challenge were respiratory disturbances.Treatment with HtE, especially at a dose of 50 mg/kg, also reduced behavioral markers of anxiety in OVA‐challenged mice. As follows from the subsequent discussion, the extract has potent anti‐inflammatory effects in the lungs, and it is thus possible that its anxiolytic effects were secondary to the improvement of the respiratory function. It is not excluded that the extract may have anxiolytic effects per se, but clarification of this question requires separate experiments.

Immunological aspects of the asthma model employed in our study are characterized by a type‐2 immune response, with a preponderance of Th2 cells. This cellular phenotype is characterized by producing cytokines such as IL‐4, IL‐5, IL‐13, and immunoglobulins (IgE/IgG1) that are considered markers of allergic asthma 37, 38, 39. Production of these immunoglobulins is dependent on IL‐4 and IL‐13 40, and high levels of IgE are associated with allergic reactions and disease severity in humans 37. The non‐sensitized animals showed no evidence of OVA‐specific IgE serum titer whereas OVA mice had high IgE titer confirming that the sensitization was effective. Treatment with HtE was able to reduce IgE titer suggesting that the extract efficiently reduced sensitization process in mice.

A classical feature of chronic inflammation in asthma is recruitment of inflammatory cells to the site of inflammation. To assess this cell migration, we performed total and differential cell counts in bronchoalveolar lavage (BAL) as well as in the lung tissue. OVA‐sensitized animals had significant increase of inflammatory cells in BAL as well as in the peribronchiolar and perivascular areas, with hyperplasia of goblet cells of the lung tissue, when compared to the Saline group. This confirmed that inflammatory process was active in the lungs of OVA‐animals. Treatment with HtE prevented inflammatory cell migration, especially eosinophils, in a dose‐dependent manner. We have previously reported that in the murine model of asthma, eosinophilia correlates with hyperreactivity and tissue damage, and that its reduction by plant extract indicates the regression of allergy symptoms 41. Reported here, HtE effect on the cellular response was comparable to the effect of dexamethasone, clearly indicating that the extract has anti‐inflammatory action.

HtE treatment was not effective in inducing increase in Treg cells in mediastinal lymph nodes suggesting that the beneficial effects of the extract arise from modulation of cell populations other than Treg. In examining the population of CD3^+^ T‐lymphocytes, we observed a significant reduction of these cells in animals treated with HtE. Similar results were demonstrated with *Cissampelos sympodialis* extract, where the reduction of CD3^+^cells in the BAL was correlated with a beneficial immunomodulation of asthma 29. In addition, HtE treatment, especially at dose of 50 mg/kg, decreased percentage of B220+ cells (B cells) in BAL. Reduction of this cell population is recognized marker of anti‐inflammatory action, once decrease of B cells also decrease immunoglobulins production.

In susceptible individuals, exposure to allergens induces a predominant profile of Th2 cytokines such as IL‐4, IL‐5, and IL‐13 38, 39. Thus, regulation of Th2 cytokines in the lungs is vital for the control of asthma symptoms. Under this assumption, we evaluated the production of IL‐13 in our experiments. The level of this cytokine was substantially elevated in sensitized animals; HtE prevented these increase, being as effective as dexamethasone (data not shown). Increased IL‐13 is associated with tissue remodeling and fibrosis by stimulating fibroblasts to synthesize collagen and also with activation of macrophages in chronic asthma. In addition, IL‐13 induces production of mucus by lung epithelial goblet cells, acting on their proliferation, differentiation, and secretory function. Another very important function of IL‐13 in allergic asthma is isotype switching to IgE by B cells 42. The HtE treatment was able to reduce this cytokine in the asthmatic mice. We also observed reduction of goblet cell hyperplasia and substantial decreased of mucus production in animals treated with the extract. This effect was as potent as the effect of dexamethasone.

Taken the immunological changes described in this study, namely the decrease by HtE treatment of OVA‐IgE serum titer, IL‐13 production and consequently inhibition of mucus production as well as inhibition of migration of inflammatory cells to the lung tissues, we conclude that the plant extract is modulating type‐2 immune response characteristic of asthma.

Therefore, our study demonstrates that *H. tiubae* extract has anti‐inflammatory and anxiolytic effects in the murine model of asthma. This corroborates the folk medicine data reporting the use of the plant to treat airway diseases. Our study does not allow concluding whether the extract possess genuine anxiolytic properties or whether anxiolytic effects were secondary to the alleviation of respiratory symptoms provoked by the airway inflammation. Another limitation of our study is lack of information regarding the content of active substances in the extract. Despite these limitations, our results suggest that *H. tiubae* extract is a promising candidate for clinical studies with the goal of developing an herbal medicine that can be included in the arsenal of anti‐asthmatic drugs.

## Conflicts of Interest

None declared.
